# Structural Basis for the Peptidoglycan-Editing Activity of YfiH

**DOI:** 10.1128/mbio.03646-21

**Published:** 2022-02-15

**Authors:** Meng-Sheng Lee, Kan-Yen Hsieh, Chiao-I Kuo, Szu-Hui Lee, Shambhavi Garde, Manjula Reddy, Chung-I Chang

**Affiliations:** a Institute of Biological Chemistry, Academia Sinica, Taipei, Taiwan; b Institute of Biochemical Sciences, College of Life Science, National Taiwan University, Taipei, Taiwan; c Centre for Cellular and Molecular Biology, Hyderabad, India; Pasteur Institute

**Keywords:** biosynthesis, editing, peptidoglycan precursor, structure

## Abstract

Bacterial cells are encased in peptidoglycan (PG), a polymer of disaccharide *N*-acetylglucosamine (GlcNAc) and *N*-acetyl-muramic acid (MurNAc) cross-linked by peptide stems. PG is synthesized in the cytoplasm as UDP-MurNAc-peptide precursors, of which the amino acid composition of the peptide is unique, with l-Ala added at the first position in most bacteria but with l-Ser or Gly in some bacteria. YfiH is a PG-editing factor whose absence causes misincorporation of l-Ser instead of l-Ala into peptide stems, but its mechanistic function is unknown. Here, we report the crystal structures of substrate-bound and product-bound YfiH, showing that YfiH is a cytoplasmic amidase that controls the incorporation of the correct amino acid to the nucleotide precursors by preferentially cleaving the nucleotide precursor by-product UDP–MurNAc–l-Ser. This work reveals an editing mechanism in the cytoplasmic steps of peptidoglycan biosynthesis.

## INTRODUCTION

Peptidoglycan (PG) is a mesh-like polymer of sugars and amino acids (aa) uniquely formed in the extracytoplasmic space of bacterial cells. The exoskeleton-like structure of PG maintains cell shape and protects the cells against high turgor pressure (1). The polymeric PG consists of linear sugar chains of alternating *N*-acetylglucosamine (GlcNAc) and *N*-acetylmuramic acid (MurNAc), connected by a β-(1,4)-glycosidic bond. Each MurNAc is attached with a tetrapeptide or a pentapeptide stem. In Escherichia coli, the stem peptide of PG contains l-alanine, d-glutamic acid, *meso*-diaminopimelic acid (*m*-DAP), and d-alanine. Cross-linking between the stem tetrapeptides, in the case of E. coli, of different sugar chains contributes to a three-dimensional mesh-like structure formation; the cross-links are normally formed between the d-alanine of one tetrapeptide and the *m*-DAP of another tetrapeptide by a peptide bond (2).

The biosynthesis of PG is initiated in the cytoplasm of bacterial cells. The cytoplasmic steps of PG biosynthesis consist of five sets of reactions, which lead to the formations of (i) UDP-GlcNAc from fructose-6-phosphate, (ii) UDP-MurNAc from UDP-GlcNAc, (iii) d-Glu from l-Glu, (iv) the dipeptide d-Ala–d-Ala from l-Ala, and (v) UDP–MurNAc–l-Ala–d-Glu–*m-*DAP–d-Ala–d-Ala (UDP-MurNAc-pentapeptide) from UDP-MurNAc (3). UDP-MurNAc-pentapeptide is assembled by sequential ligations of l-Ala, d-Glu, *m*-DAP, and d-Ala–d-Ala to UDP-MurNAc, which are ATP-dependent reactions catalyzed by four amino acid ligases, MurC, MurD, MurE, and MurF, respectively (3). Once synthesized, UDP-MurNAc-pentapeptide is then transferred to the inner side of the cytoplasmic membrane to form lipid-anchored intermediates, of which the final product, lipid II, is transported to the outer side of the cytoplasmic membrane to be used by the PG synthases and transpeptidases for polymerization and cross-linking.

The specificities of the Mur ligases are not absolute; for example, although the preferred substrate of MurC is l-Ala, l-Ser or Gly can be added with lower efficiencies *in vitro* (4, 5). It has been shown that the MurC ligases isolated from two bacterial species with different amino acids at the first position of the PG-peptide stem exhibit similar substrate specificities (6). A previous work has shown that YfiH (also known as PgeF) is a PG-editing factor with a role in maintaining a specific PG composition in E. coli (7). The absence of *yfiH* leads to incorporation of l-Ser into the first position of the stem peptide, which is normally occupied by l-Ala, resulting in β-lactam sensitivity, altered cell morphology, and reduced PG synthesis (7). Here, we elucidate the molecular mechanism of YfiH. We report the crystal structures of YfiH bound to a trapped endogenous UDP-MurNAc, as well as to the preferred substrate UDP–MurNAc–l-Ser (UMS). Our results show that YfiH forms an extended L-shaped binding groove for the UDP-MurNAc-monopeptide and is a cytoplasmic amidase specific for hydrolyzing UDP–MurNAc–l-Ser, a noncanonical reaction by-product from the MurC reaction. This work suggests that bacteria possess a cytoplasmic precursor-editing mechanism to maintain the specific amino acid composition of PG.

## RESULTS

### Overall structure of YfiH bound to UDP-MurNAc.

**FIG 1:**
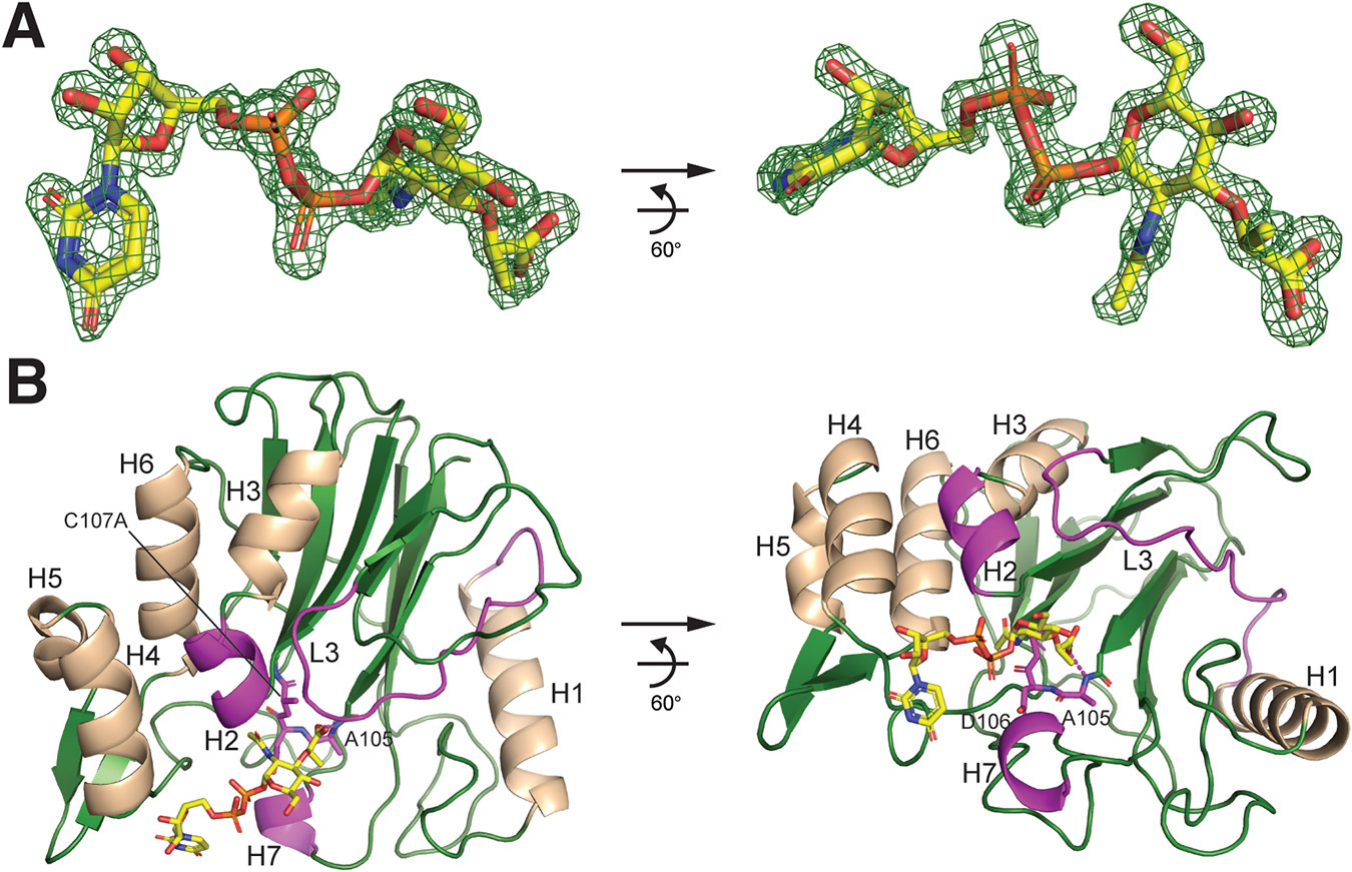
Structure of UDP-MurNAc bound to YfiH-C107A. (A) *Fo-Fc* difference Fourier omit map of UDP-MurNAc, shown as sticks and in two views, at a 1.47-Å resolution displayed in green isomesh at a 4.0 σ level. (B) Ribbon diagram of the structure of YfiH, shown in forest green and in two views, with bound UDP-MurNAc as sticks. Helices are colored in wheat. The two helices and the loop interacting with the compound are in magenta. The catalytic β-turn is shown as magenta sticks.

We expressed catalytically inactive recombinant E. coli YfiH-C107A protein for the original purpose of screening potential substrates or ligands by binding assays and cocrystallography. Purified YfiH-C107A forms highly diffracting crystals; the best crystal diffracted to a 1.47-Å resolution in the space group *P*2_1_2_1_2_1_, with four molecules (chains A to D) per asymmetric unit (see Table S1 in the supplemental material). Chains A and C contain almost all of the YfiH amino acid residues except for Met1, 2 to ∼243; chains B and D contain residues 3 to ∼243. In chains C and D, residues 81 to 84 and 79 to 85, respectively, from a solvent-exposed loop are disordered. The presence of the N-terminal region in the structure indicates that YfiH does not possess a signal peptide and therefore is a cytoplasmic protein. Serendipitously, the electron density map contained a prominent blob of well-resolved density for an endogenous compound, presumably copurified with YfiH-C107A and trapped during crystallogenesis. This compound was unambiguously identified as UDP-MurNAc based on its high-resolution map (Fig. 1A). The description and presentation of the complex structure here are based on chain A unless mentioned otherwise.

10.1128/mbio.03646-21.6 TABLE S1 Crystallographic data collection and refinement statistics. Download Table S1, DOCX file, 0.01 MB. Copyright © 2022 Lee et al. 2022 Lee et al. https://creativecommons.org/licenses/by/4.0/ This content is distributed under the terms of the Creative Commons Attribution 4.0 International license.

YfiH is conserved in Gram-negative bacteria, in most Gram-positive bacteria except for a couple of species (7), and in vertebrates (Fig. S1). The structure of UDP-MurNAc-bound E. coli YfiH adopts an overall globular structure that features a pair of two central β-sheets sandwiched by multiple α-helices (Fig. 1B and Fig. S2), which is similar to the structures of Shigella flexneri YfiH and Geobacillus stearothermophilus YlmD (with root mean square difference [RMSD] values of 0.9 Å and 1.3 Å, respectively) (8, 9). UDP-MurNAc is bound to a preformed groove located on one side of the double β-sheets and at a distinct β-turn (aa 104 to 108). The β-turn bridges the two β-sheets; importantly, it harbors the catalytic Cys107 and forms the oxyanion hole by the backbone amides of Ala105 and Asp106 (Fig. 1B). The groove is demarcated by two short helices, H2 and H7, and an extensive loop, L3 (Fig. S2), which together guard the catalytic β-turn.

10.1128/mbio.03646-21.1 FIG S1 Phylogenetic tree of YfiH proteins from selected representative species of diverse phyla. Phylogenetic analysis of YfiH and its homologs in bacteria and vertebrates listed by genus names. The three groups are indicated by circles with different colors. The UniProt or GenBank accession numbers are provided in parentheses. The maximum-likelihood tree was constructed by using MEGA 11 software (K. Tamura, G. Stecher, and S. Kumar, Mol Biol Evol 38:3022–3027, 2021, https://doi.org/10.1093/molbev/msab120); the distance of each node is shown by the branch lines. The YfiH homologs of Gram-positive bacteria are divided into many subclades due to their sequence diversity. The labeled nodes with black circles contain the sequences chosen for further sequence analysis, shown in Fig. S2. Download FIG S1, TIF file, 2.4 MB. Copyright © 2022 Lee et al. 2022 Lee et al. https://creativecommons.org/licenses/by/4.0/ This content is distributed under the terms of the Creative Commons Attribution 4.0 International license.

10.1128/mbio.03646-21.2 FIG S2 Secondary structure of YfiH and sequence alignment with YlmD and the homologous C-terminal domain (CTD) of human FAMIN/Lacc1. Secondary-structure elements of YfiH are indicated above the alignment. α-Helices and β-strands are illustrated by magenta and blue rectangles; loops are represented by green lines. Catalytic Cys residues are highlighted in a shaded box. Binding residues of YfiH for the moieties of UDP, MurNAc, and monopeptide of the substrate are encircled in red, orange, and gray boxes, respectively. YlmD- and FAMIN-CTD-specific insertion sequences are in cyan and brown, respectively. The UniProt IDs of the aligned sequences are as follows: P33644 for E. coli YfiH, P84138 for Geobacillus stearothermophilus YlmD, and Q8IV20 for human FAMIN/Lacc1. Download FIG S2, TIF file, 1.8 MB. Copyright © 2022 Lee et al. 2022 Lee et al. https://creativecommons.org/licenses/by/4.0/ This content is distributed under the terms of the Creative Commons Attribution 4.0 International license.

### Interaction of YfiH with UDP-MurNAc-monopeptide.

**FIG 2:**
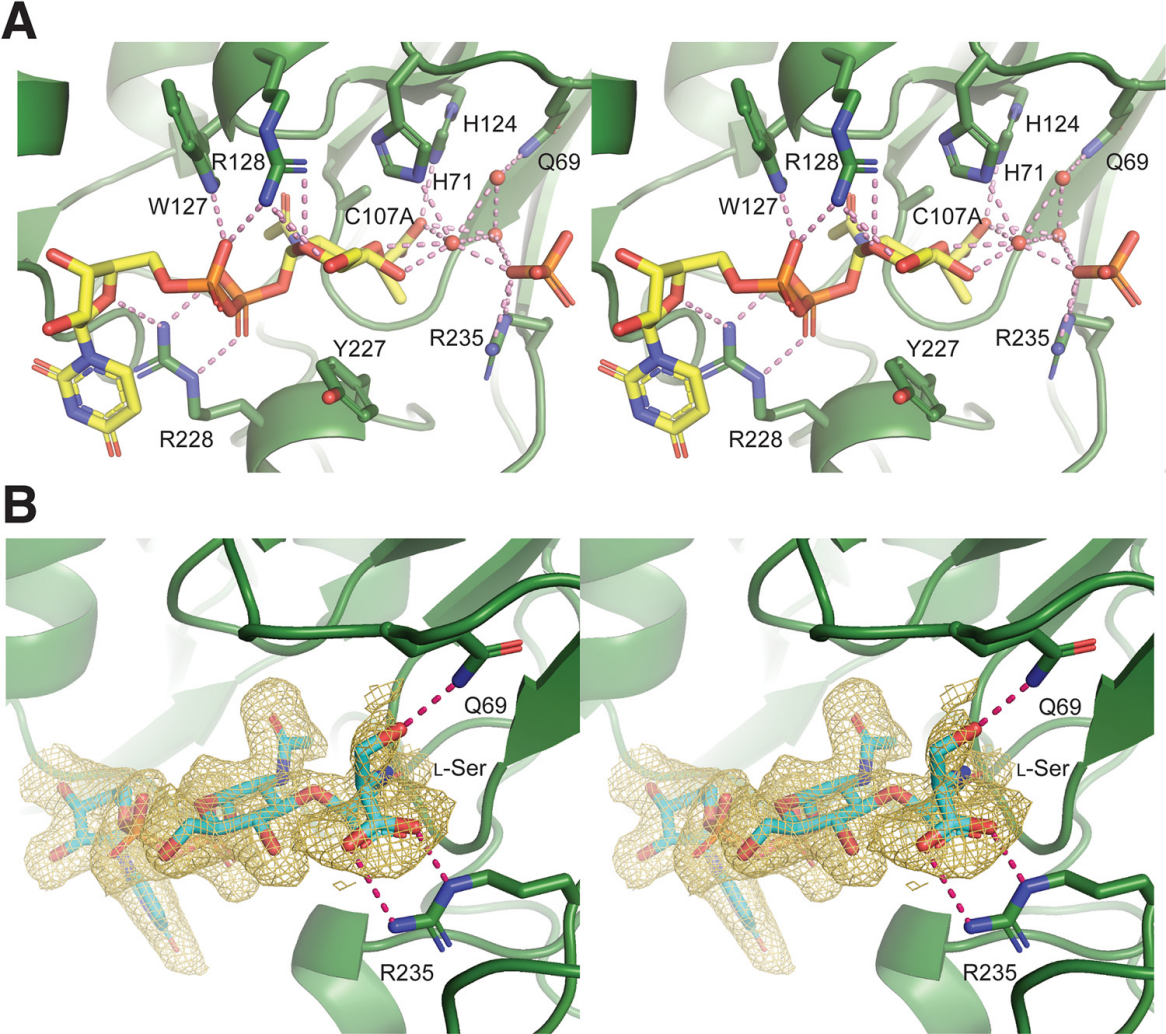
Interactions of UDP-MurNAc and UDP–MurNAc–l-Ser with the binding groove in YfiH. (A) Stereoview of the bound UDP-MurNAc and the phosphate ion in the complex structure with YfiH-C107A. Water oxygen atoms are shown as spheres. (B) The *Fo-Fc* difference Fourier omit map of UDP–MurNAc–l-Ser bound to YfiH-C107A is displayed in a gold isomesh at a 1.5 σ level in stereoview. The compounds and the side chains of the interacting residues are shown as sticks. Hydrogen bonds are shown as dashed bonds.

UDP-MurNAc forms a largely extended conformation in the elongated L-shaped binding groove of YfiH, where the uridine moiety forms a sharp angle with the diphosphate-linked MurNAc (Fig. 2A). The aromatic ring of uracil packs against the side chain of Arg228 from H7, forming the turning point of the groove. Notably, Arg228 interacts not only with the uracil ring but also with the ribose ring oxygen and the diphosphate of UDP. The UDP diphosphate also makes electrostatic contacts with Trp127 and Arg128 from H2. The sugar ring of MurNAc is stacked with Tyr227 from H7. The lactyl oxygens of MurNAc interact with His71 from L3 and His124, which forms a catalytic triad with Asp89 and Cys107.

Interestingly, in two of the four YfiH molecules (chains A and B) in the asymmetric unit, a bound phosphate ion is found ∼4.9 Å away from the lactic acid moiety of MurNAc and connected to the free carboxyl group via a hydrogen-bonding network involving several ordered water molecules (Fig. 2A). The crystallographic structure of these solvent molecules bound to the MurNAc residue strongly suggests the presence of a binding pocket for an amino acid and two putative interacting residues, Gln69 and Arg235, which coordinate the water and the phosphate ions, respectively. Therefore, the substrate of YfiH may be a UDP-MurNAc-monopeptide. Based on the genetic observation that the absence of *yfiH* leads to the misincorporation of l-Ser instead of l-Ala into PG, our crystallographic results suggest that YfiH may prevent the incorporation of l-Ser by specifically hydrolyzing UDP–MurNAc–l-Ser (UMS) as the preferred substrate.

To test this idea, we obtained the crystals of YfiH bound to UDP–MurNAc–l-Ser, which was synthesized enzymatically (described below), by washing and soaking the YfiH-UDP-MurNAc crystals with an excess amount of the compound. The cocrystal structure, determined at a 1.86-Å resolution (Table S1), shows that Gln69 indeed forms a hydrogen bond with the side chain hydroxyl of l-Ser of the substrate, whose carboxylic group forms a bidentate salt bridge with Arg235 (Fig. 2B).

### UMS is the preferred substrate of YfiH.

**FIG 3:**
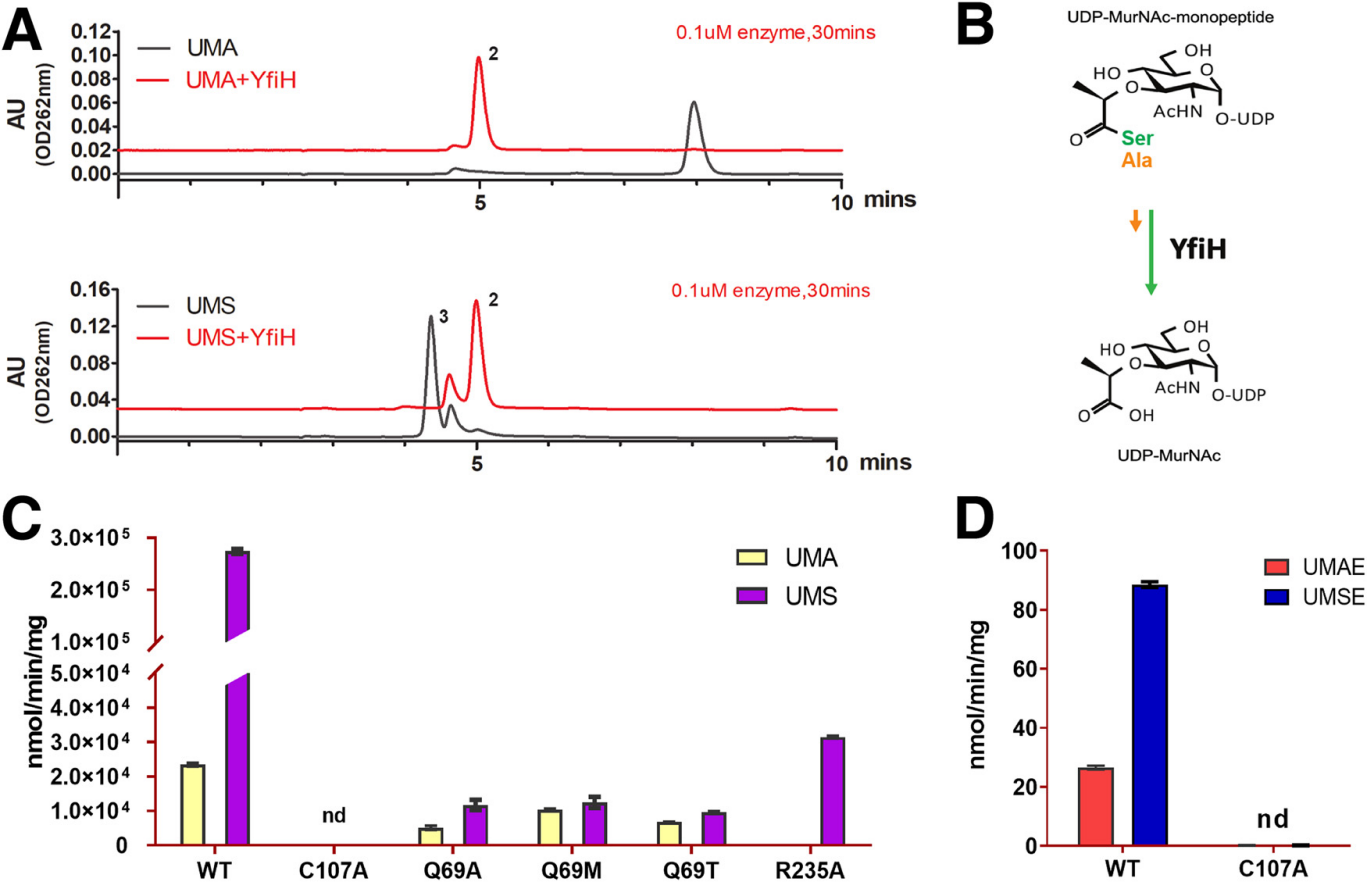
Hydrolyzing activities of YfiH on UDP-MurNAc-peptides and mutational analysis. (A) HPLC chromatograms of UDP–MurNAc–l-Ala (UMA; compound 1) and UDP–MurNAc–l-Ser (UMS; compound 3) before and after incubation with YfiH to yield the cleavage product UDP-MurNAc (compound 2). AU, arbitrary units. (B) Hydrolytic reaction catalyzed by YfiH and the substrate preference for UDP–MurNAc–l-Ser rather than UDP–MurNAc–l-Ala. (C) Histogram showing the comparison of the specific activities of wild-type YfiH (WT) and mutants on UMA and UMS. (D) Histogram showing the comparison of the specific activities of wild-type YfiH and the inactivated C107A mutant on UMAE (UDP–MurNAc–l-Ala–γ-d-Glu) and UMSE (UDP–MurNAc–l-Ser–γ-d-Glu). nd, not determined.

We sought to test whether YfiH specifically hydrolyzes UDP–MurNAc–l-Ser into UDP-MurNAc, as well as to determine the role of Gln69 in recognition specificity. To this end, we synthesized UDP–MurNAc–l-Ala (UMA) and UMS using recombinant MurC; based on the purified UDP-MurNAc-monopeptides, we further synthesized UDP–MurNAc–l-Ala–d-Glu (UMAE) and UDP–MurNAc–l-Ser–d-Glu (UMSE) with recombinant MurD. Purification and quantitative determination of the compounds were performed using high-performance liquid chromatography (HPLC) and verified by mass spectrometry (see Materials and Methods). We also purified UDP–MurNAc–l-Ala–d-Glu–*m*-DAP (UM-Tri) using an established method (10). As expected, wild-type YfiH hydrolyzes UDP-MurNAc-monopeptide into UDP-MurNAc (Fig. 3A and B); the hydrolytic activity requires the catalytic residue Cys107 (Fig. 3C and D). Moreover, the measured specific activity of YfiH on UMS was more than 10-fold greater than on UMA (Fig. 3C and Table 1). Removing the side chain of Arg235 significantly decreased the hydrolytic activity (Fig. 2B and 3C). Interestingly, YfiH also hydrolyzed chemically synthesized MurNAc–l-Ser (Fig. S3A). Furthermore, we found that YfiH also cleaves the UM dipeptides UMAE and UMSE (Fig. S3B and C). The substrate preference is maintained toward UMSE, although with significantly lower specific activities (Fig. 3C and D). Lastly, we could not detect the activity of YfiH on UM-Tri (Fig. S3D). Taken together, these results demonstrate that YfiH is a cytoplasmic hydrolase specific for the PG precursor UDP–MurNAc–l-Ser.

**TABLE 1:** Enzyme activities of YfiH mutants with different UDP-MurNAc derivatives

Phenotype	Substrate	Activity (nmol/min/mg)
WT	UMA	23,451 ± 474
	UMS	274,084 ± 4521
	UMAE	27 ± 1
	UMSE	89 ± 1

C107A	UMA	ND^*a*^
	UMS	ND
	UMAE	ND
	UMSE	ND

Q69A	UMA	5,117 ± 583
	UMS	11,747 ± 1,490

Q69M	UMA	10,353 ± 228
	UMS	12,489 ± 1,633

Q69T	UMA	6,756 ± 52
	UMS	9,544 ± 192

R235A	UMS	31,495 ± 98

a ND, not detected.

10.1128/mbio.03646-21.3 FIG S3 Hydrolyzing activities of YfiH on various UDP-MurNAc-peptide derivatives. (A) HPLC chromatograms of MurNAc–l-Ser (MS; compounds 1′ and 1″ with MurNAc in the α and β anomeric forms, respectively) before and after incubation with YfiH, yielding the cleavage product MurNAc (compounds 2′ and 2″ in the α and β anomeric forms, respectively). (B) HPLC chromatograms of UDP–MurNAc–l-Ala–γ-d-Glu (UMAE; compound 3) before and after incubation with YfiH, yielding the cleavage product UDP-MurNAc (compound 4). (C) HPLC chromatograms of UDP–MurNAc–l-Ser–γ-d-Glu (UMSE; compound 5) before and after incubation with YfiH, yielding the cleavage product UDP-MurNAc (compound 4). (D) HPLC chromatograms of UDP–MurNAc–l-Ala–γ-d-Glu–*m*-DAP (UMTri; compound 6) before and after incubation with YfiH. Download FIG S3, TIF file, 1.3 MB. Copyright © 2022 Lee et al. 2022 Lee et al. https://creativecommons.org/licenses/by/4.0/ This content is distributed under the terms of the Creative Commons Attribution 4.0 International license.

We engineered two YfiH mutants with Q69A and Q69M mutations to compare their specific activities on the various substrates. The results show that the activity for UMS, but not for UMA, was significantly reduced in both the Q69A and Q69M mutants and that the activity for UMA was affected in the Q69A mutant (Fig. 3C and D). Therefore, the long polar side chain of Gln69 may be responsible for interacting with the amino acid moiety of the UDP-MurNAc-monopeptide. Overall, these results demonstrate that Gln69 is the specificity determinant residue.

**FIG 4:**
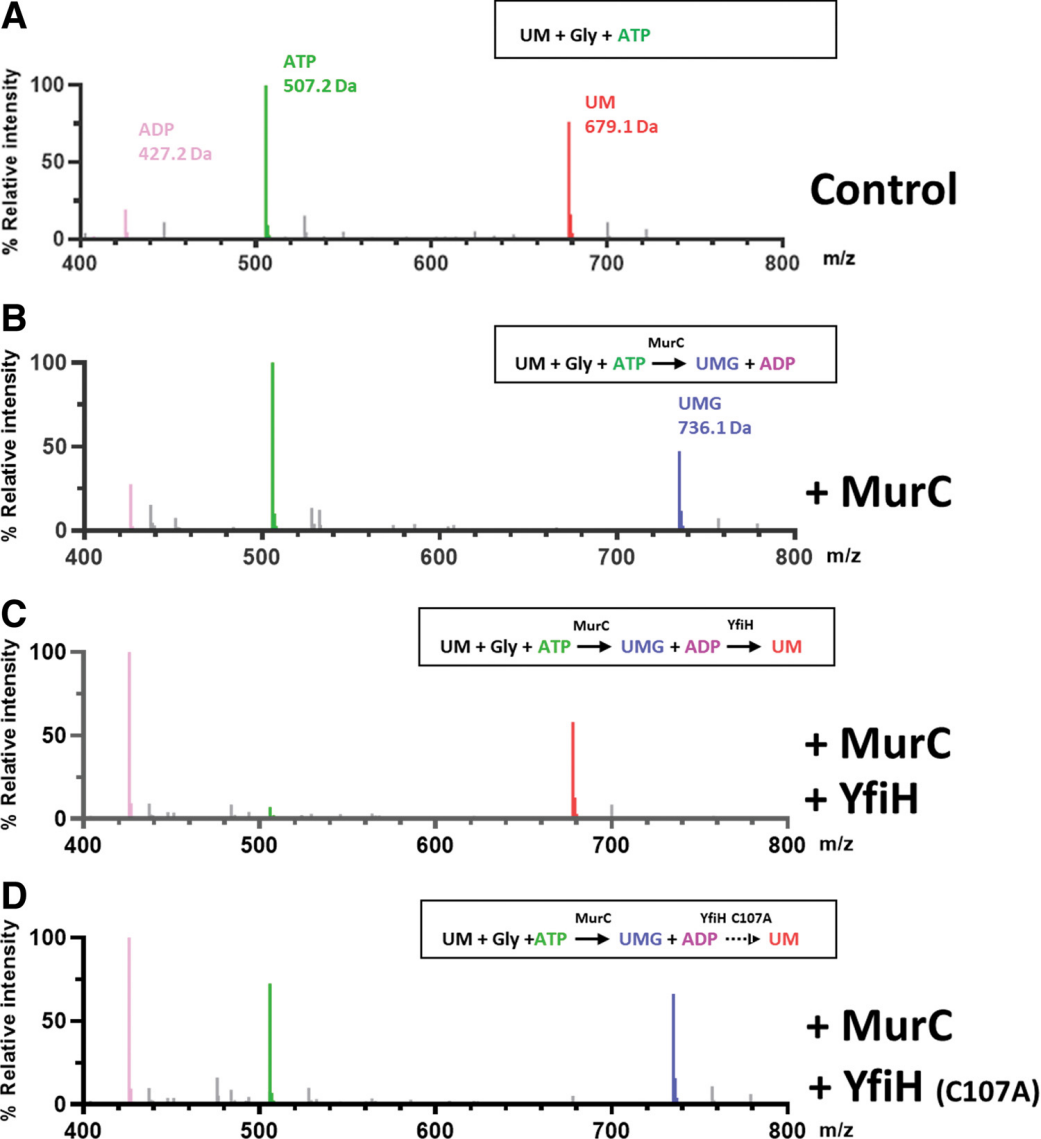
YfiH also hydrolyzes UDP-MurNAc-Gly. (A to D) Mass spectra of UDP-MurNAc-Gly (UMG; 736.1 Da) after a 24-h reaction of 1 mM UDP-MurNAc (UM; 679.1 Da), 5 mM ATP, and 18 mM Gly without (A) or with (B) 20 μM MurC, coincubation with 20 μM MurC and 10 μM wild-type YfiH (C), or coincubation with 20 μM MurC and 10 μM YfiH-C107A (D).

We have also tested the hydrolytic activity of YfiH on UDP-MurNAc-Gly (UMG) since a previous genetic study suggested that YfiH also prevents the incorporation of glycine-containing muropeptides (7). We attempted to prepare UMG by enzymatic synthesis with MurC, using glycine as a substrate; however, the product yield was very low, and purified UMG was extremely unstable in aqueous solution. Therefore, we developed an alternative assay in which mass spectrometry was used to examine the effect of YfiH on the synthesis of UMG by MurC in the presence of limited amounts of the substrates UM, glycine, and ATP. If YfiH is able to hydrolyze UMG, in the MurC reaction, it will prevent the accumulation of the product UMG. Concurrently, ATP, which is consumed by the MurC reaction, will be exhausted, and eventually no UMG will be present in the reaction mixture. The mass spectra indeed showed that the UMG peak yielded from the MurC reaction disappeared when YfiH, but not YfiH-C107A, was included in the reaction mixture (Fig. 4A to C).

## DISCUSSION

**FIG 5:**
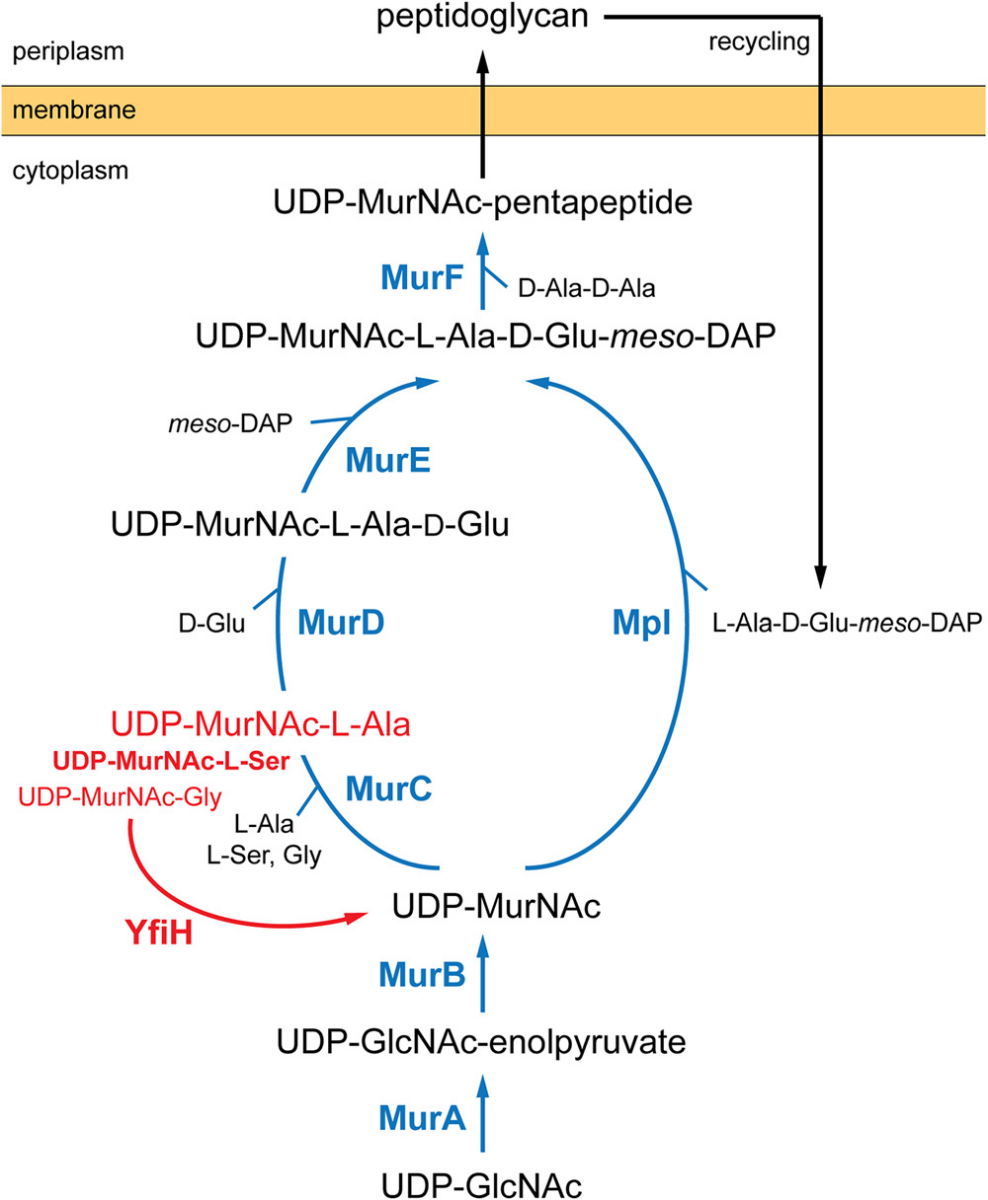
Involvement of YfiH in the cytoplasmic steps of peptidoglycan biosynthesis and recycling. The reactions catalyzed by the ATP-dependent Mur/Mpl ligases are in blue. The reactions catalyzed by YfiH are in red.

In this work, we show that YfiH is involved in a previously unknown cytoplasmic editing mechanism for the biosynthesis of PG (Fig. 5). We show that YfiH hydrolyzes UDP-MurNAc-monopeptides, which are synthesized by MurC, into UDP-MurNAc and amino acids. The preferred substrate of YfiH is UMS, against which the specific activity of YfiH is more than 10-fold higher than that of UMA. Although we were unable to determine the specific activity for UMG due to its poor stability, it is likely that YfiH hydrolyzes UMG at a rate comparable, if not inferior, to the rate for UMA due to the lack of a side chain for Gly. The hydrolytic reactions catalyzed by YfiH ensure the incorporation of the specific amino acid l-Ala into the cytoplasmic UDP precursors of PG. l-Ala is the first amino acid of the peptide moiety of PG in nearly all eubacteria (11). In some bacterial species, however, l-Ser or Gly is added at this position instead of l-Ala (11). In the stepwise assembly of monomeric precursors of PG, the formation of the amide bond between UDP-MurNAc and the first amino acid is catalyzed by the MurC ligase (3). Interestingly, all purified MurC ligases from bacteria whose first amino acid of the PG-peptide is l-Ala or Gly exhibit preferred catalysis for the synthesis of UDP–MurNAc–l-Ala over UDP-MurNAc-Gly (4, 6). E. coli MurC can take l-Ala, l-Ser, or Gly as the substrate; the *K_m_* values for l-Ala, l-Ser, and Gly were 20, 850, and 2,500 μM, respectively (4). Intriguingly, the MurC ligases from Mycobacterium leprae and Mycobacterium tuberculosis, which contain Gly and l-Ala in the first position of the stem peptide, respectively, showed similar *K_m_* and *V*_max_ values for l-alanine and glycine (6). These results suggest that the presence of the first species-specific amino acid in the stem peptide of PG is achieved by a mechanism not explained by the substrate specificity of MurC. Our results showing YfiH as an l-Ser-specific UDP-MurNAc-monopeptide amidase therefore uncover a bacterial control mechanism to ensure only the presence of the specific amino acid in the peptide assembly pathways by removing unwanted UDP-MurNAc-monopeptide byproducts from the MurC reactions.

It is likely that bacteria rely on specific YfiH-like hydrolases to maintain a PG with specific stem peptides. However, it remains to be seen whether bacterial cells can regulate the expression of YfiH in response to environmental stimuli to alter the composition of the first amino acid of the PG stem peptide, thereby evading the attack of muralytic enzymes to gain a survival advantage. E. coli mutant cells without YfiH activity are hypersensitive to several β-lactam antibiotics (7). The structures of YfiH bound to the substrate and product presented in this study may facilitate the development of specific inhibitors, which may be used in combination with certain β-lactam antibiotics to achieve synergistic therapeutic benefits.

YfiH belongs to a family of proteins containing domain of unknown function 152 (DUF152), classified in the Pfam database. Our structural and functional analysis of E. coli YfiH uncovers a binding groove for the PG precursor UDP-MurNAc-monopeptide. Interestingly, YfiH also cleaves MurNAc–l-Ser (Fig. S3A). This result suggests that the conserved L-shaped binding groove formed in the structure of DUF152 proteins may bind extracytoplasmic PG-related fragments, which may be mechanistically important for the function of some homologs found in higher eukaryotic organisms. One possibility is that some of the DUF152 proteins may have lost enzymatic activity but evolved to retain specific ligand-binding activity, as seen in the peptidoglycan recognition proteins (PGRPs), which play sensor roles in innate immune recognition (12). FAMIN, a DUF152 protein, is shown to be overexpressed in macrophages under muramyl dipeptide (MDP) treatment and associated with NOD_2_-induced intracellular microbial clearance (13). Based on a homology model of FAMIN–C-terminal domain (CTD) predicted by AlphaFold, we have noticed that in the conserved binding groove, the specificity determinant residue is a threonine (Thr74) in lieu of glutamine; moreover, the location of the arginine likely interacting with the carboxylic group of the amino acid moiety of the PG precursor is different from that of Arg235 of YfiH, which may be essential for catalytic activity (Fig. 2 and Fig. S4). The misplacement of the substrate carboxyl group-coordinating basic residue and the change of the specificity determinant residue in FAMIN-CTD suggest that it may function as a sensor recognizing UDP–MurNAc–l-Ala or MurNAc–l-Ala. Alternatively, FAMIN-CTD may have hydrolyzing activity on muramyl dipeptide fragments. Future studies will be needed to characterize the enzymatic or binding activities of these DUF152 proteins and their biological roles.

10.1128/mbio.03646-21.4 FIG S4 Comparison of the structure of YfiH and a homology model of FAMIN/Lacc1. (A) Structure of E. coli YfiH bound to UDP-MurNAc. The loop region showing a major structural difference from FAMIN is in magenta. (B) Homology model of FAMIN-CTD, predicted by AlphaFold, in an orientation similar to that of YfiH. The FAMIN-specific insertion is highlighted in brown. (C) Superimposition of the structures of YfiH and FAMIN-CTD. The bound compound and the interacting residues are shown as sticks. The catalytic Cys residue is indicated by the red label or the asterisk. The putative binding residues for the amino acid moiety of UDP-MurNAc-monopeptide are indicated by the dashed boxes. Download FIG S4, TIF file, 2.9 MB. Copyright © 2022 Lee et al. 2022 Lee et al. https://creativecommons.org/licenses/by/4.0/ This content is distributed under the terms of the Creative Commons Attribution 4.0 International license.

## MATERIALS AND METHODS

### Cloning and mutagenesis.

All plasmids in this study were subcloned into a pET21a(+) vector with a C-terminal 6×His tag. YfiH-C107A, YfiH-Q69A, and YfiH-Q69M were generated using the wild-type YfiH (UniProtKB accession no. P33644) plasmid, reported previously (7), as the template by PCR-based site-directed mutagenesis. The genes encoding MurC (UniProtKB accession no. P17952) and MurD (UniProtKB accession no. P14900) were cloned from competent E. coli BL21(DE3) cells by PCR. All constructs in this study were sequenced prior to use by the DNA Sequencing Core Facility of the Academia Sinica (AS-CFII-108-115).

### Protein expression and purification.

The plasmid was transformed into E. coli BL21(DE3) ECOS cells (Yeastern Biotech). Cells were cultured to an optical density at 600 nm (OD_600_) of 0.6 to 0.8 and induced with 1 mM isopropyl β-d-thiogalactopyranoside at 20°C for 18 h. Cell pellets were resuspended in lysis buffer containing 50 mM Tris-HCl, pH 8.0, 500 mM NaCl with protease inhibitor cocktail (Roche) and then ruptured by a French press (Avestin). After centrifugation at 35,000 × *g* at 4°C for 45 min, the supernatant was applied to a nickel-nitrilotriacetic acid agarose (Qiagen) column and washed with 20 mM imidazole twice. The protein fraction eluted with 250 mM imidazole was purified further by MonoQ 5/50 GL column chromatography (GE Healthcare) at pH 8.0, and a Superdex 200 10/300 GL column (GE Healthcare) was equilibrated in 20 mM Tris-HCl, pH 8.0, 100 mM NaCl, and 1 mM dithiothreitol (DTT).

### Crystallization, structure determination, and refinement.

The crystallization of YfiH-C107A was performed by using the sitting-drop vapor diffusion method at 22°C, in which 1 μL of a 15-mg/mL protein solution was mixed with 1 μL of reservoir solution consisting of 2.0 M ammonium sulfate and 100 mM sodium acetate at pH 5.4. The crystals of YfiH-C107A bound to UDP-MurNAc-Ser were prepared by washing the YfiH-C107A crystals, which contain bound endogenous UDP-MurNAc, in 10 μL of the mother liquid twice, and then the crystals were transferred to a 5-μL drop of freshly prepared reservoir solution consisting of 2.2 M ammonium sulfate and 100 mM sodium phosphate at pH 5.4, to which 0.25 μL of 20 mM UDP-MurNAc-Ser was added, and incubated for 1.5 weeks. The procedure was repeated twice. The crystals were cryoprotected by a brief transfer to the mother liquid supplemented with 10 to 30% xylitol prior to data collection.

All diffraction data were collected on NSRRC beamline 15A1 (NSRRC Taiwan). All images were indexed, integrated, and scaled using the HKL-2000 package (14). The structures were solved by molecular replacement with the program PHASER (15). The crystal structure of Escherichia coli YfiH (Protein Data Bank accession no. 1Z9T) was used as the initial search model. Structure refinement and manual modeling were implemented using the programs Refmac5 and COOT, respectively (16, 17). Native ligands bound in crystal structures were explored using the program LigandFit in Phenix. The cutoff minimum correlation coefficient was set to 0.75 to avoid uncorrected ligand placement. All protein model figures were generated with PyMOL (v.1.7.2; Schrödinger).

### Synthesis of UDP-MurNAc-peptide derivatives.

UDP-MurNAc-Ala and UDP-MurNAc-Ser were produced by enzymatic synthesis. The processes were performed in a reaction mixture containing 20 mM Tris-HCl, pH 8.0, 0.5 mM UDP-MurNAc (Chiralix), 5 mM ATP, 10 mM MgCl_2_, and 0.01 mM MurC enzyme, followed by the addition of 1 mM l-alanine or l-serine as the amino acid donor. The reaction was performed completely at 37°C for 18 h and then stopped by removing enzymes with an Amicon Ultra-15 centrifugal filter unit (Millipore). The product was purified by reverse-phase HPLC. The fraction with the desired UDP-MurNAc amino acid was collected at 262 nm and reconfirmed by electrospray ionization-mass spectrometry (ESI-MS). The qualified fractions were lyophilized and reconstituted in deionized water. The synthesis of UDP-MurNAc-Ala-Glu or UDP-MurNAc-Ser-Glu was as described above, but we used extra 2 mM d-glutamate and 0.5 mM MurD enzyme during the reaction step.

### Procedures to synthesize the MurNAc-Ser derivatives.

All reactions were conducted in oven-dried glassware under a nitrogen atmosphere (Fig. S5). The reaction products were purified by using column chromatography on silica gel (Geduran silica gel 60, 0.040 to 0.063 mm), a Buchi Puro850 automated purification machine, or HPLC. Anhydrous solvents and moisture-sensitive materials were transferred by using an oven-dried syringe or cannula through a rubber septum. Organic solutions were concentrated under reduced pressure in a water bath (<40°C). Thin-layer chromatography (TLC) was performed on precoated glass plates of TLC silica gel 60G F254 (Merck KGaA), and cells were detected with a UV lamp (254 nm) and/or by staining reagents that contained ceric ammonium molybdate (for general use), *p*-anisaldehyde (for sugars), or ninhydrin (for amine or amide). ^1^H nuclear magnetic resonance (NMR) spectra were recorded on Bruker AVII-500 (500 MHz) spectrometers by using CD_3_OD (chemical shift value [*d_H_*] = 3.31 ppm, central line of a quintet) or D_2_O (*d_H_* = 4.80 ppm) as internal standards. High-resolution mass spectrometry (HRMS) was performed on Bruker Bio-TOF III (ESI-TOF) spectrometers, and results are reported as mass/charge (*m/z*) ratios with the percentage relative abundance. The solvents for extraction and chromatography were of American Chemical Society (ACS) grade. Anhydrous *N*,*N*-dimethylformamide (DMF) was purchased from Aldrich Chemical Co. in a sealed package and stored in an electronic dry box. *N*-Acetylmuramic acid (MurNAc), *N*-hydroxysuccinimide (NHS), *N*,*N*-diisopropylcarbodiimide (DIC), H-Ser-OtBu HCl salt, triethylamine (TEA), triisopropylsilane (TIS), and trifluoroacetic acid (TFA) were purchased from Bachem, BLD, TRC, BLD, J.-T. Baker, Aldrich, and Alfa Aesar Chemical Co., respectively. All the chemicals were directly used without further purification unless otherwise specified.

10.1128/mbio.03646-21.5 FIG S5 Procedures to synthesize the MurNAc-Ser derivatives. DIC (80 μL, 0.51 mmol) was dropwise added into the solutions of MurNAc (100 mg, 0.34 mmol) and NHS (47 mg, 0.41 mmol) in dry DMF (20 mL) with stirring at 0°C under a nitrogen atmosphere. The product, NHS ester-activated MurNAc, was further used for the next step. To the MurNAc-NHS ester (79 mg, 0.20 mmol) and H-Ser-OtBu HCl salt (80 mg, 0.40 mmol), TEA (112 μL, 0.80 mmol) was dropwise added at 0°C under a nitrogen atmosphere with stirring and further purified to obtain the desired MurNAc-Ser-OtBu (43 mg, 49% yield). The MurNAc-Ser-OtBu (6 mg, 14 μmol) then reacted with 200 μL of acid recipe TFA-H_2_O-TIS (95/2.5/2.5, vol/vol/vol) to synthesize the target compound, MurNAc-Ser-OH (0.5 mg, 10% yield). Download FIG S5, TIF file, 0.2 MB. Copyright © 2022 Lee et al. 2022 Lee et al. https://creativecommons.org/licenses/by/4.0/ This content is distributed under the terms of the Creative Commons Attribution 4.0 International license.

### Enzyme activity assay.

In the assay, 3 nM YfiH enzymes were incubated with 10 μM UDP-muropeptide derivatives in a reaction buffer containing 20 mM Tris-HCl, pH 8.0, and 2 mM DTT; the final volume in each assay was adjusted to 50 μL, and then the mixture was incubated in 37°C for 15 min, except under the conditions of wild-type YfiH with UDP-MurNAc-Ser, which was incubated for 7 min due to unexpectedly high activity. All reactions were paused immediately by freezing the mixtures in liquid nitrogen until HPLC detection.

The efficiency of substrate hydrolysis in each reaction was quantified by monitoring the change of initial peak areas under 262 nm by HPLC. Peak areas for the analyte were proportional to the injected amount and were confirmed at initial assay establishment. Fraction eluates were further analyzed by mass spectrometry to address the identities of final reaction products in assays.

### ESI-MS analysis.

The experiments aiming for high resolution and high mass accuracy in this study were done on an LTQ Orbitrap XL ETD mass spectrometer (Thermo Fisher Scientific) equipped with a standard ESI ion source. Five-microliter samples were flow injected at a rate of 50 μL min^−1^ in 80% acetonitrile (ACN)-H_2_O-0.1% formic acid (FA) by the Acquity ultrahigh-performance LC (UPLC) system from Waters (Waters). The full-scan MS condition was a mass range *m/z* 200 to 2,000 and a resolution of 60,000 at *m/z* 400. The electrospray voltage was maintained at 4 kV, and the capillary temperature was set at 275°C.

### HPLC.

The experiments of reverse-phase HPLC in this study were done on a Waters Alliance 2695 separation module (Waters) with a Hypersil base-deactivated silica (BDS) C_18_ column (Thermo Fisher Scientific) and μBondapack C_18_ column (Waters). Samples were injected into the column with an isocratic flow of 50 mM ammonium formate, pH 4.3, at 1 mL min^−1^. Quantification of the peak area was analyzed with Empower 3 data chromatography software (Waters).

### Data availability.

The structural factors and coordinates have been deposited in the Protein Data Bank under the accession codes 7F3V and 7W1G for the complexes of YfiH with YfiH-UDP-MurNAc and YfiH-UDP–MurNAc–l-Ser, respectively.
