# Supplementary material for: Structural Basis for the Peptidoglycan-Editing Activity of YfiH
Source: mBio. 2022 Feb 15;13(1):e03646-21. doi: 10.1128/mbio.03646-21 (PMC8844914; doi:10.1128/mbio.03646-21)
Supplement: TABLE S1 [file mbio.03646-21-st001.docx]

**Table S1. Crystallographic data collection and refinement statistics**

|  | **YfiH-C107A** |  |
| --- | --- | --- |
| **Data collection** |  |  |
| Wavelength (Å) | 1.00 |  |
| Space group | P212121 |  |
| Cell dimensions |  |  |
| *a*, *b*, *c* (Å) | 69.95, 98.83, 136.24 |  |
| *α*, *β*, *γ* (°) | 90, 90, 90 |  |
| Resolution (Å) | 50-1.47 (1.52-1.47)^a^ |  |
| I/σI | 40.08 (2.4) |  |
| R_merge_ | 0.033 (0.529) |  |
| R_pim_^b^ | 0.017 (0.27) |  |
| CC_1/2_^c^ | 0.965 (0.854) |  |
| Completeness (%) | 97.3 (94.0) |  |
| Redundancy | 6.9 (6.7) |  |
| **Refinement** |  |  |
| Resolution (Å) | 34.08-1.47 (1.51-1.47) |  |
| No. of reflections | 141889 (7130) |  |
| Reflections used for R_free_ | 7531 (356) |  |
| *R*_work_ | 0.185 (0.255) |  |
| *R*_free_ | 0.214 (0.28) |  |
| No. atoms |  |  |
| Protein | 7297 |  |
| Water | 838 |  |
| B-factors (Å^2^) |  |  |
| Protein | 16.88 |  |
| Water | 26.32 |  |
| R.m.s. deviations |  |  |
| Bond lengths (Å) | 0.013 |  |
| Bond angles (º) | 1.857 |  |
| Validation |  |  |
| Clash score | 4 |  |
| Rotamer outliers (%) | 1.74 |  |
| Ramachandran plot |  |  |
| Favored/Allowed/Disallowed (%) | 96.2/3.3/0.5 |  |

^a^ Highest resolution shell is shown in parenthesis.

^b^*R*_pim_ is the precision-indicating merging *R*, which describes the accuracy of the averaged measurement [(1)](https://paperpile.com/c/fJWtvZ/lrpfe).

^c^CC^1/2^ is the correlation coefficient between two random half data sets [(2)](https://paperpile.com/c/fJWtvZ/SFPn).

**References:**

1. [Weiss MS. 2001. Global indicators of X-ray data quality. Journal of Applied Crystallography.](http://paperpile.com/b/fJWtvZ/lrpfe)

2. [Karplus PA, Diederichs K. 2012. Linking crystallographic model and data quality. Science 336:1030–1033.](http://paperpile.com/b/fJWtvZ/SFPn)
