# Supplementary figures and images for: Structural Basis for the Peptidoglycan-Editing Activity of YfiH
Source: mBio. 2022 Feb 15;13(1):e03646-21. doi: 10.1128/mbio.03646-21 (PMC8844914; doi:10.1128/mbio.03646-21)

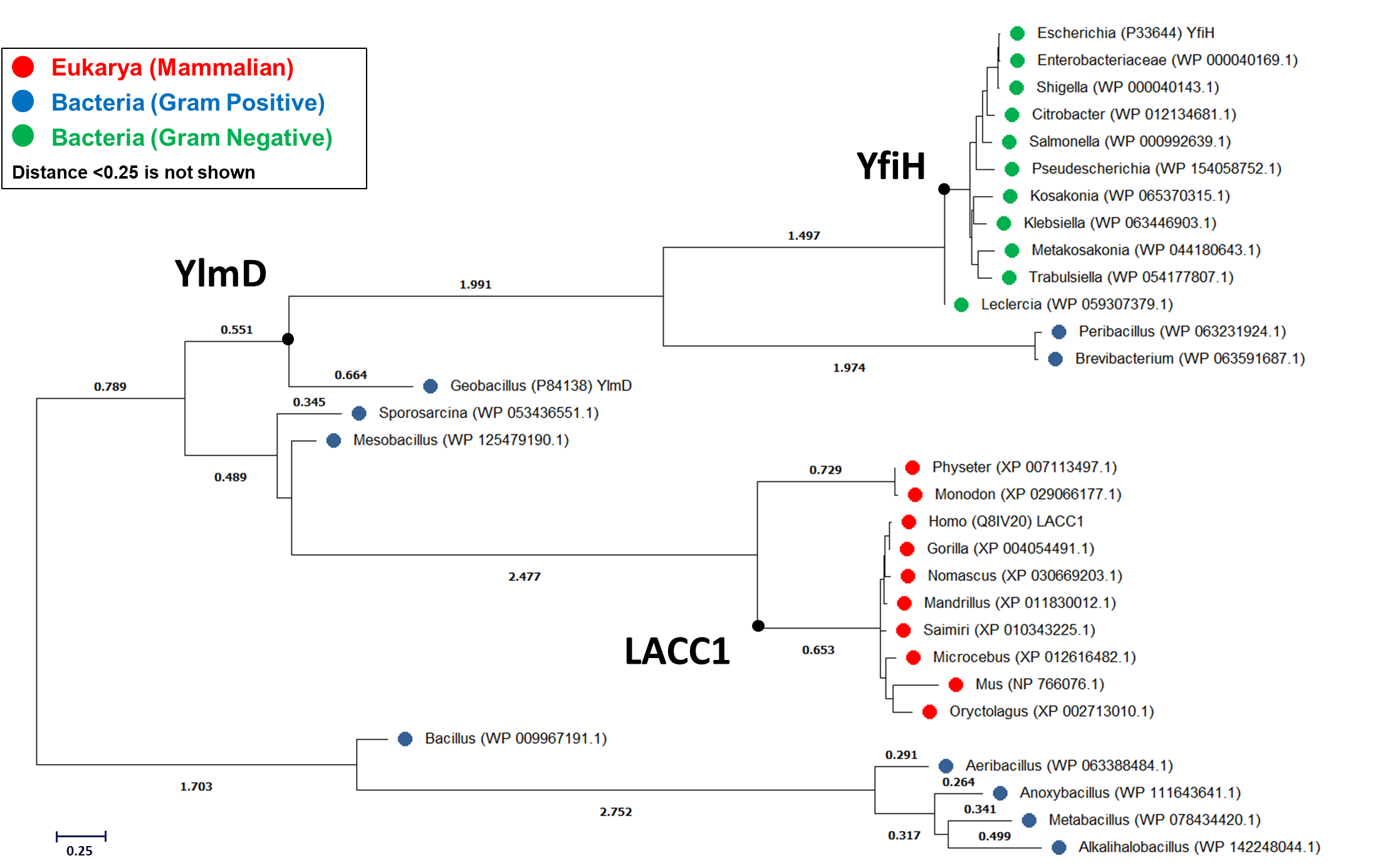

Supplement: FIG S1 [file mbio.03646-21-sf001.tif]

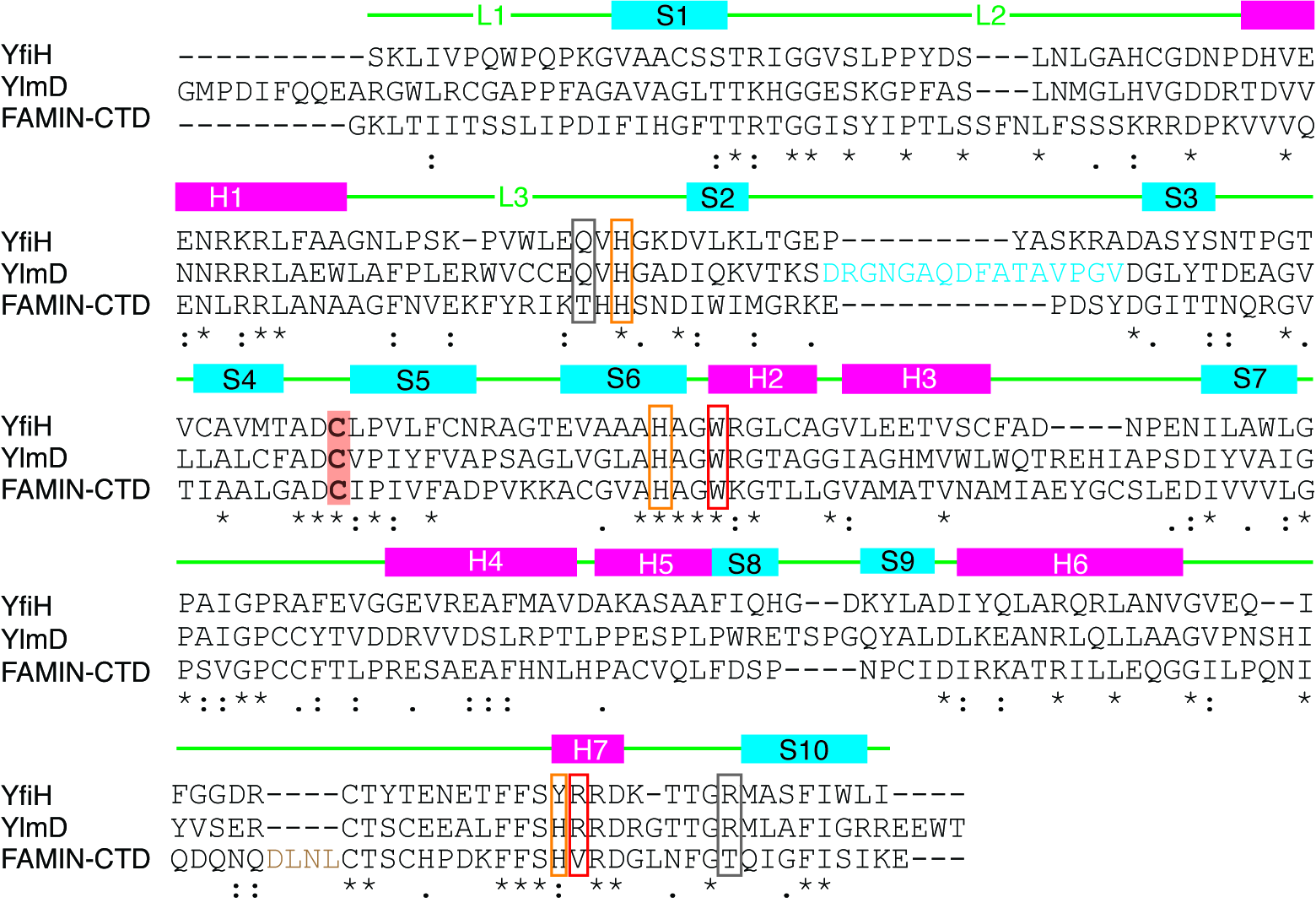

Supplement: FIG S2 [file mbio.03646-21-sf002.tif]

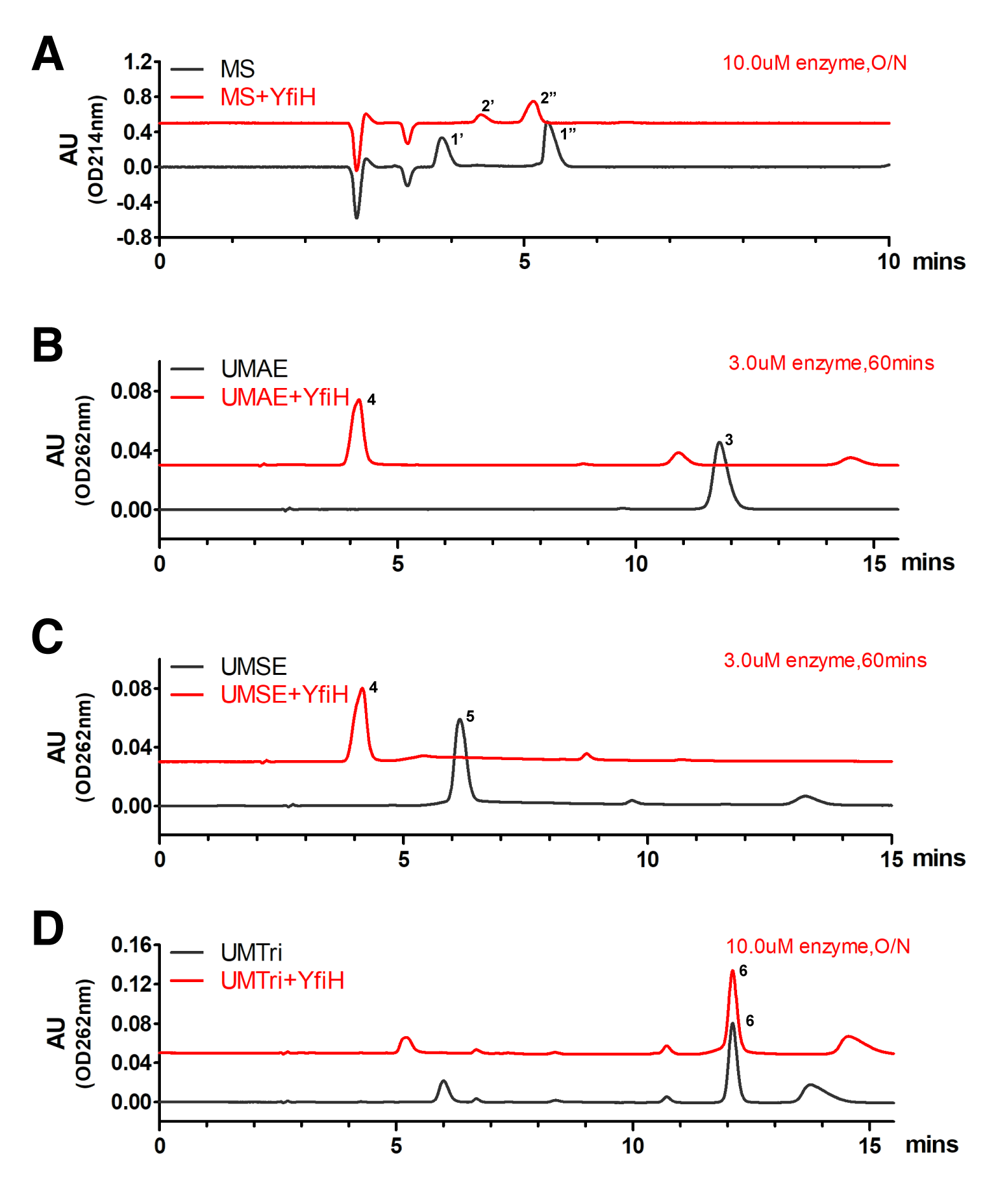

Supplement: FIG S3 [file mbio.03646-21-sf003.tif]

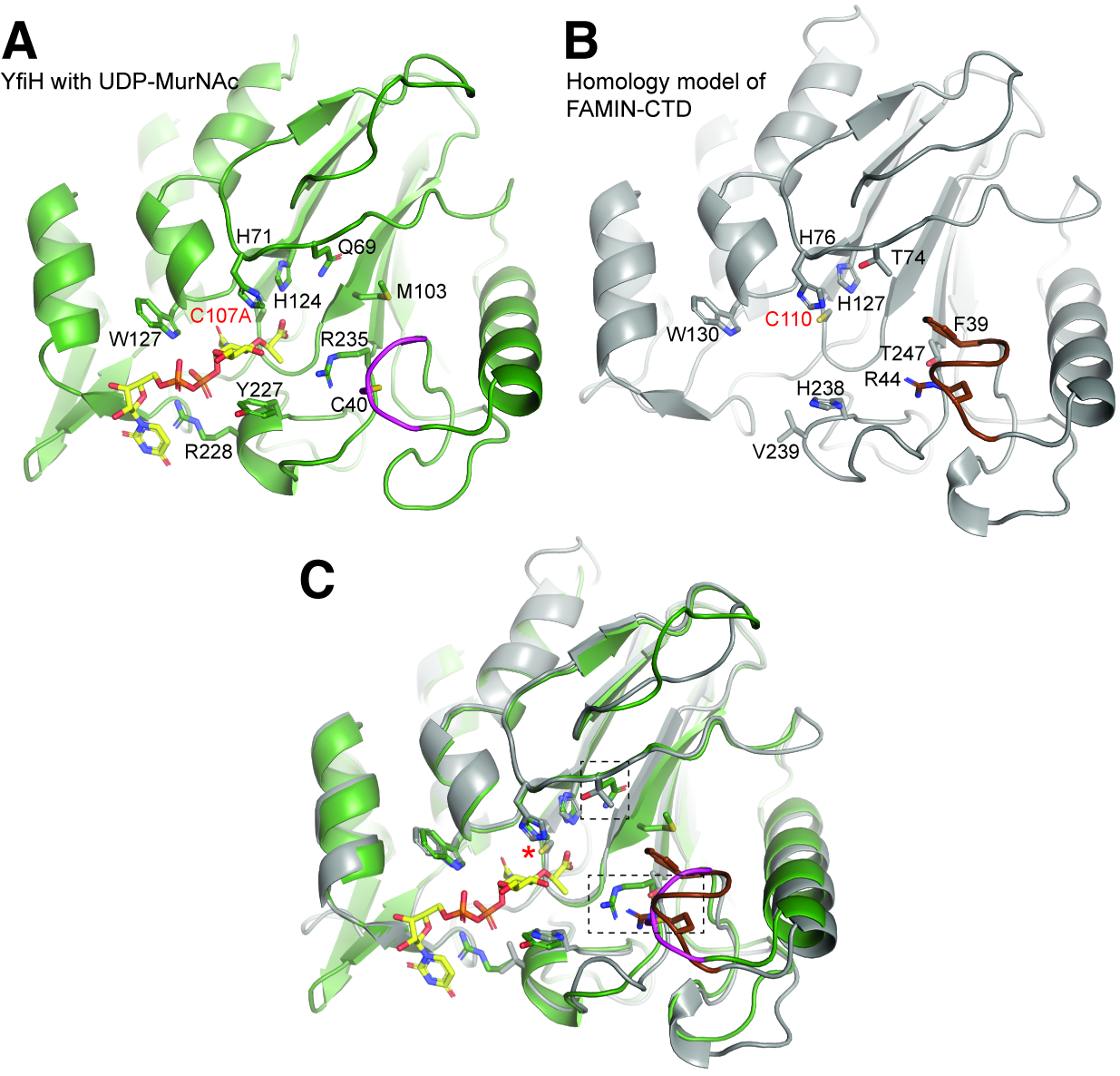

Supplement: FIG S4 [file mbio.03646-21-sf004.tif]

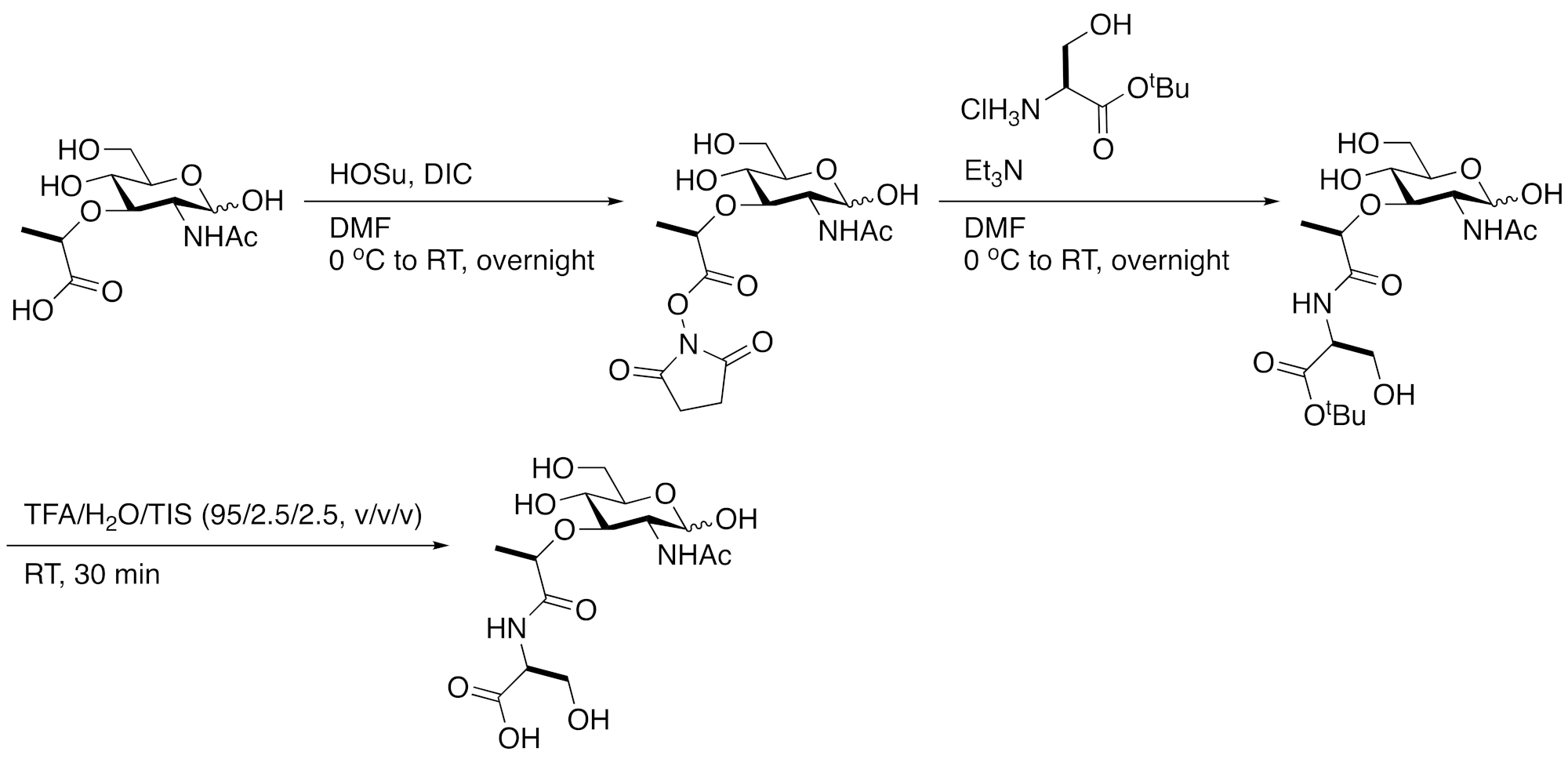

Supplement: FIG S5 [file mbio.03646-21-sf005.tif]
